# Association of Weight Loss from Early to Middle Adulthood and Incident Hypertension Risk Later in Life

**DOI:** 10.3390/nu12092622

**Published:** 2020-08-28

**Authors:** Yunping Zhou, Tao Wang, Xin Yin, Yun Sun, Wei Jie Seow

**Affiliations:** 1 School of Nursing, Qingdao University, No. 308 Ningxia Road, Qingdao 266071, China; lwzhouyunping@163.com; 2 School of Public Health, Qingdao University, Qingdao 266071, China; 2018010056@qdu.edu.cn; 3 Saw Swee Hock School of Public Health, National University of Singapore and National University Health System, Singapore 117549, Singapore; yinxin@u.nus.edu; 4 Friedman School of Nutrition Science and Policy, Tufts University, Boston, MA 02111, USA; yun.sun@tufts.edu; 5 Department of Medicine, Yong Loo Lin School of Medicine, National University of Singapore and National University Health System, Singapore 119228, Singapore

**Keywords:** body mass index, hypertension, cohort study

## Abstract

Background: The effect of obesity in early adulthood and weight loss on incident hypertension in older age has not been well characterized. This study aimed to examine the association of weight loss from young adulthood to midlife with risk of incident hypertension later in life. Methods: We performed a retrospective cohort study using data from the National Health and Nutrition Examination Survey (NHANES). Five weight change groups were categorized: stable normal, weight loss, weight gain, maximum overweight and stable obese. The hazard ratios (HRs) and 95% confidence intervals (CIs) of the association between weight change and risk of hypertension in later life were estimated using Cox regression models. Results: Compared with participants who maintained normal weight, the stable obese, weight gain, maximum overweight and weight loss groups exhibited significantly higher risks of incident hypertension, with HR of 3.28 (95% CI = 2.71 to 3.96), 2.93 (95% CI = 2.62 to 3.28), 1.76 (95% CI = 1.55 to 2.00) and 1.97 (95% CI = 1.17 to 3.31), respectively. We also observed a lower risk among those in the weight loss group (HR = 0.60, 95% CI = 0.35 to 1.02) compared with those who were stable obese. Conclusions: Weight loss from early to middle adulthood was associated with lower risk of incident hypertension as compared to those who stayed obese and higher risk of incident hypertension as compared to those who maintained normal weight. Thus, maintaining normal weight throughout adulthood may be important for the primary prevention of hypertension.

## 1. Introduction

Hypertension or high blood pressure is a major risk factor for cardiovascular diseases and contributes substantially to the increasing trend in morbidity and premature death [1,2]. The prevalence of hypertension remained high among US adults from 1999 to 2018, and the age-adjusted prevalence of hypertension among adults aged 18 and over was 45.4% in 2017–2018 [3]. It is largely known that obesity is strongly and independently associated with the incidence of hypertension [4,5,6]. Several epidemiologic studies have evaluated the association between weight change across childhood and risk of developing hypertension. The National Longitudinal Study of Adolescent Health in US assessed the relationship between weight change from adolescence to young adulthood and risk of hypertension, and reported an increased risk of hypertension for those who gained weight from adolescence to young adulthood [7]. Both the Nurses’ Health Study (NHS) and the Health Professionals Follow-Up Study (HPFS) have shown that long-term weight gain across early to middle adulthood was associated with adverse health outcomes including hypertension [8].

However, data on the association between weight loss from early adulthood to midlife and incident hypertension in later life were limited. Excess adiposity tends to accrue during early and midlife for most people [9]. In addition, previous evaluations of the association between weight change and incident hypertension have been limited to samples that are not nationally representative. The relative effect of obesity in early adulthood and weight loss on the incidence of hypertension in older age was not well characterized [10]. We sought to estimate the association between weight loss from young adulthood into middle age and risk of developing hypertension later in life with a national representative sample.

The National Health and Nutrition Examination Survey (NHANES) [11] has routinely collected information about participants’ history of weight (weight at age 25 and weight at 10 years before the survey) and hypertension diagnosis. In this study, we used these data [12,13] to evaluate the association between weight change from early-mid adulthood and hypertension incidence. We aimed to evaluate whether obese participants who lost weight were (1) at a lower risk of hypertension when compared to participants who were stable obese, and (2) at a higher risk of hypertension when compared to participants who remained stable normal.

## 2. Materials and Methods

### 2.1. Study Design and Population

NHANES is a series of ongoing cross-sectional surveys conducted by the National Center for Health Statistics (NCHS) of the Centers for Disease Control and Prevention (CDC). Representative samples of the non-institutional US population were selected by a complex stratified, multistage probability sampling design. NHANES was approved by the National Center for Health Statistics research ethics review board, and written informed consent from all the participants was provided during the survey. Details on the study design, protocol, and data collection methods have been documented [14].

This study used data across all nine cycles of the continuous NHANES (1999–2000 through 2015–2016), including adults aged 40–74 at the time of NHANES survey. Weight change was self-reported by participants of their weights at age 25 and 10 years before the NHANES survey. We defined baseline as the time 10 years before the NHANES survey. Incident hypertension was identified from participants confirming a diagnosis of hypertension by a healthcare provider. The reported age at hypertension diagnosis was used as the time of hypertension onset. The study design is shown in Figure S1. The NHANES study design has been described in detail previously [12,15].

We excluded participants who were younger than 40 years or older than 74 years, those without diagnosis information of hypertension, those without diagnosis time and those without BMI at age 25 years and (or) at baseline age. We also excluded prevalent hypertension cases who reported a date of onset that was before the initiation of 10-years follow-up. An individual who did not report a diagnosis of hypertension but the mean of three blood pressure measurements met hypertension criteria (mean systolic blood pressure ≥140 mm Hg, mean diastolic blood pressure ≥90 mm Hg) at the NHANES survey examination was considered to have undiagnosed hypertension and was thus excluded from the main analyses. Finally, a sample size of 14,542 individuals remained for analysis (Figure S2).

### 2.2. Assessments of Weight Change and Covariates

Participants were asked to recall their weight at age 25 and 10 years before the NHANES survey. Measured height from the NHANES examination was used to calculate BMI. However, self-reported height at age 25 was used to calculate BMI at age 25, and measured height was used to calculate baseline BMI for participants who were 50 years or older at the time of the survey. BMI change categories were used to capture weight change of each participant over time. Based on BMI (kg/m^2^) at age 25 and BMI at baseline [12,15], five BMI change categories were used: stable normal (BMI_age 25_ < 25 and BMI_baseline_ < 25), maximum overweight (25–29.9 at either time but not ≥30 at the other time), non-obese to obese (<30 at BMI_age 25_ and ≥30 at BMI_baseline_), obese to non-obese (≥30 at BMI_age 25_ and <30 at BMI_baseline_), and stable obese (≥30 at both times). In order to draw comparison with other studies [8,15], absolute weight change across the two time intervals were also classified into five groups: weight loss ≥2.5 kg (reference group), weight change within 2.5 kg, 2.5 kg ≤ weight gain <10.0 kg, 10 kg ≤ weight gain < 20.0 kg, and weight gain ≥ 20.0 kg.

The potential confounding factors including sex, age at baseline, race/ethnicity, family income, educational level and smoking status were obtained via the demographic questionnaires.

### 2.3. Statistical Analysis

To account for the complex survey design, we conducted all analyses using appropriate sample weights, strata, and cluster variables [16]. Descriptive statistics for all characteristics were computed using means and standard deviation (SD) for continuous variables and percentages for categorical and binary variables. Cox proportional hazards regression analyses were performed to estimate the hazard ratio (HR) and corresponding 95% CI of hypertension risk associated with weight change across young adulthood and midlife. The proportional hazards assumption was tested by comparing survival curves for weight change patterns. We found no violations of the proportionality assumption.

In the main analysis, we evaluated the relationship between the five weight change categories and risk of incident hypertension. To assess the reduced risk, all weight-change categories were compared to the stable obese category (reference). To assess the increased risk, all weight-change categories were compared to the stable normal category (reference). The first model (model 1) was adjusted for sex, baseline age (years, continuous), and race/ethnicity (non-Hispanic White, non-Hispanic Black, Mexican American, and others). The second model (model 2) additionally adjusted for family income–poverty ratio level (≤1.3, ~1.85, ~3.0, and >3.0), education level (less than high school, high school or equivalent, and college or above), and smoking status (never and ever smoker). Stratified analyses and potential effect modifications were conducted by baseline age (<50 and ≥50 years) and sex. Multiplicative interaction was calculated by including cross-product interaction terms in the multivariable Cox regression models.

For the absolute weight change analyses, BMI at 25 years was also included as a potential confounder in model 2. We further evaluated the relationship between absolute weight change patterns and hypertension risk, as well as the possible linear dose–response relationship. For test of trend, we calculated the association with incident hypertension by treating the categories of absolute weight change as ordinal variables. Furthermore, we analyzed the association between absolute weight change groups and incident hypertension risk stratified by BMI at 25 years of age.

Sensitivity analyses were conducted by including participants who did not report a diagnosis of hypertension but the mean of three blood pressure measurements met hypertension criteria (mean SBP ≥ 140 mm Hg, mean DBP ≥ 90 mm Hg) at examination to test the robustness of the results.

All statistical analyses were performed using SAS version 9.4 (site 70239492). Statistical significance was defined as *p* < 0.05 using two-sided tests.

## 3. Results

### 3.1. Baseline Characteristics and Weight Change Pattern

The characteristics of study participants are presented in Table 1. The mean baseline age was 42.4 years, and 51.8% were female. The mean BMI was 23.3 kg/m^2^ at age 25, and 26.5 kg/m^2^ at baseline. Most of the participants were non-Hispanic White (74.2%). Overall, from early to middle adulthood, 44.0% of the participants remained stable normal, 4.9% remained stable obese, 15.1% reported gaining weight, and only 0.9% reported losing weight from the obese to non-obese category.

### 3.2. Associations of Weight and Weight Change Patterns with Incident Hypertension

**Figure 1:**
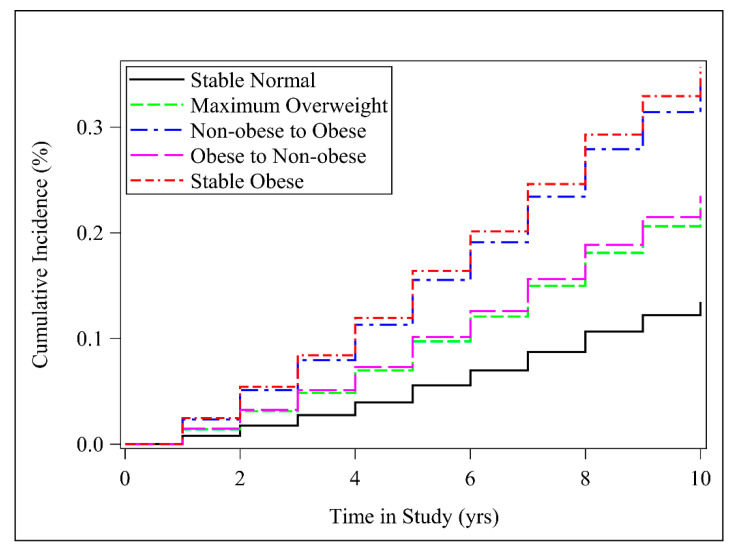
Cumulative incidence curve for hypertension by weight change category. Multivariable Cox regression model adjusted for sex, baseline age, race/ethnicity, family income, education, and smoking status. Stable normal (BMI < 25.0 at both timepoints), maximum overweight (BMI 25.0–29.9 at either timepoint but not ≥30.0 at the other timepoint), non-obese to obese (BMI < 30.0 at younger age and ≥30.0 later), obese to non-obese (BMI ≥ 30.0 at younger age and <30.0 later), and stable obese (BMI ≥ 30.0 at both timepoints).

When evaluating the weight status at baseline (Table S1), we found that overweight and obesity were significantly associated with incident hypertension. There was a significant dose–response analysis between obesity at baseline and hypertension risk (*p*_for trend_ < 0.001). Table 2 shows the association between weight change patterns across early adulthood and incident hypertension risk, using the stable normal group and the stable obese group as the reference, respectively. During an average of 10 years follow-up and 132,640 person-years, 3177 cases of incident hypertension were identified; the incidence rate was 23.95 per 1000 person-years. Figure 1 presents cumulative incidence curves by time in study for each weight change pattern.

Compared with the stable normal category, the stable obese group had higher risk of developing hypertension, with HR (95% CI) of 3.28 (2.71 to 3.96). Weight gain from the non-obese range to the obese range was associated with a higher risk of hypertension (HR = 2.93, 95% CI = 2.62 to 3.28). Individuals who reported losing weight from the obese category to the non-obese group had 1.97 (95% CI = 1.17 to 3.31) times the risk of developing incident hypertension. The maximum overweight group also showed a significant association with hypertension risk, with HR (95% CI) of 1.76 (1.55 to 2.00).

Compared with the stable obese group (Table 2), the stable normal group had the lowest risk (HR = 0.31, 95% CI = 0.25, 0.37) of developing hypertension. Furthermore, both gaining weight from the non-obese range and losing weight from the obese category were associated with a lower risk of hypertension (HR = 0.89, 95% CI = 0.73 to 1.09; HR = 0.60, 95% CI = 0.35 to 1.02, respectively). The maximum overweight category also indicated a significant association, with HR (95% CI) of 0.54 (0.44 to 0.65).

**Figure 2:**
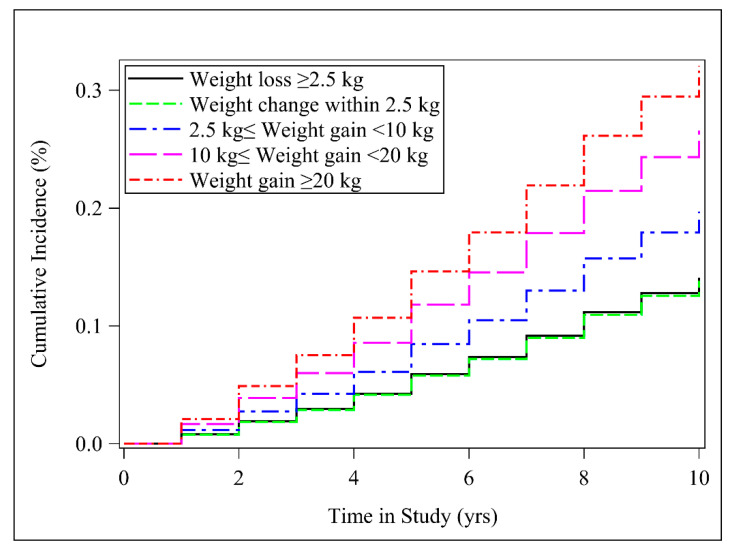
Cumulative incidence curve for hypertension by the absolute weight change category. Multivariable Cox regression model adjusted for sex, baseline age, race/ethnicity, family income, education, smoking status, and BMI at 25 years.

We identified a significant linear dose–response association for incident hypertension with weight change (*P*_for trend_ < 0.001). When classified into categories, the HRs (95% CI) for hypertension in the extreme weight gain group (weight gain ≥ 20 kg), moderate to large weight gain (10 kg ≤ weight gain < 20 kg) and small to moderate weight gain (2.5 kg ≤ Weight gain < 10 kg) were 2.64 (2.07 to 3.37), 2.09 (1.67 to 2.60), and 1.46 (1.14 to1.87), respectively, when compared to the weight loss group (weight loss ≥ 2.5 kg) (Table 3). Figure 2 presents cumulative incidence curves by time in study for each absolute weight change group. Furthermore, we restricted the analysis to participants who had a BMI of ≥25 at 25 years of age, and the results remained similar (Table S2).

**Figure 3:**
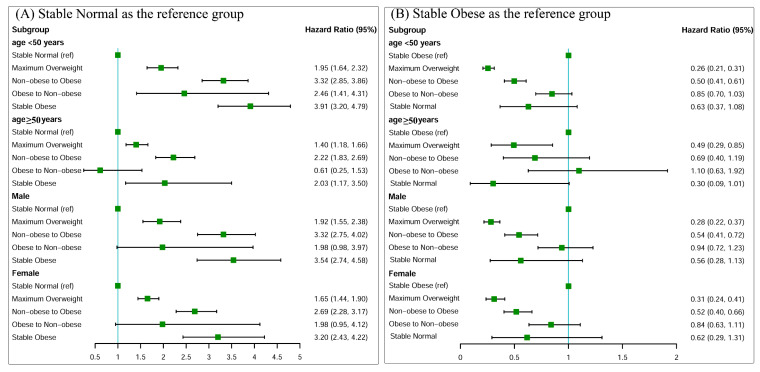
Associations between weight change patterns across adulthood and risk of incident hypertension stratified by baseline age, sex. (**A**) Stable normal as the reference group; (**B**) stable obese as the reference group. Risk estimates were adjusted for sex (not adjusted in stratified analysis by sex), baseline age (not adjusted in stratified analysis by age), race/ethnicity, family income, education, and smoking status.

Associations between weight change patterns across early adulthood and risk of incident hypertension stratified by baseline age and sex are shown in Figure 3. Compared with the stable normal category, we found significant interactions with age. All interaction *p* values for age and weight change patterns were <0.05. Stronger associations were observed among participants who were less than 50 years old as compared with their older counterparts. 

To test the stability of our results, we performed sensitivity analyses after inclusion of those who had elevated blood pressure at examination, and the results were almost unchanged (Table S3).

## 4. Discussion

In this large retrospective cohort study of nationally representative US adults, we found that weight loss (obese to non-obese) from early to midlife had reduced risk of developing hypertension when compared with the stable obese group. Nonetheless, weight loss was associated with a higher hypertension risk compared with stable normal group. In addition, stable obese, maximum overweight and weight gain from early to midlife were all associated with higher risks of incident hypertension when compared with stable normal participants. Moreover, a significant linear dose–response association was identified for incident hypertension risk with absolute weight change, regardless of their weight at early adulthood. These findings underscored the importance of maintaining normal weight across adulthood for lowering hypertension risk in later life.

The relationship between weight gain and hypertension has been extensively evaluated in many prospective studies [7,17,18,19]. The Johns Hopkins Precursors Study [20] including 1183 men observed that individuals with normal weight in young adulthood but became overweight or obese in middle adulthood were twice more likely to develop hypertension than men who remained stable normal weight. Another recent study [21] by Hou et al. assessed the association between weight change from childhood to early adulthood and adult hypertension risk in the Chinese population, and showed that weight gain was associated with increased incident risk of hypertension with a HR of 3.75 (95% CI = 2.49 to 5.64) for normal weight in childhood but overweight/obese in early adulthood. The current study tried to capture the changes in BMI during adulthood before the hypertension was developed and to minimize reverse causation. In this study, we reached a consistent conclusion with evidence based on other non-national sources that suggest that stable obesity and weight gain across young adult and midlife are associated with greater incident hypertension risk.

Among obese individuals, our study found that weight loss was associated with lower risk of hypertension compared with those who were stable obese. The relatively small sample size (only 0.9% of the participants switched from obese to non-obese from young to middle adulthood) may be the reason that we detected a marginal significant association between obese to non-obese participants [22]. In the Nurses’ Health Study, the researchers reported that the benefit from weight loss was substantially limited to women who had a higher BMI at baseline [23]. Future prospective studies that investigate the association of weight change (intentional and unintentional) with hypertension risk are warranted to clarify the interpretations of our findings. Weight loss has major public health implications among overweight or obese adults for the primary prevention of hypertension. Therefore, maintaining a normal weight throughout adulthood may be important for the primary prevention of hypertension, and may be adequate to achieve the global target of 25% relative reduction of hypertension incidence [24]. This study also implied that the risk of incident hypertension was higher (HR = 1.97, 95% CI = 1.17 to 3.31) if an individual with obesity in early adulthood changed to normal weight in midlife, suggesting the importance of weight control in young adulthood.

As the participants’ ages (40–74 years) spanned over a wide range, there is a large variation of time period between age 25 and baseline [25]. Participants were then stratified by baseline age. Evidently, positive associations between weight change and hypertension risk were consistently found in all the baseline age groups, suggesting that the association between weight change and hypertension risk remained irrespective of the time period between age 25 and baseline. Subgroup analyses by sex indicated that the associations between BMI change and incident hypertension were similar in males and females. The distribution of ethnicity was different among the weight change patterns, and we also found that the incidence of hypertension in non-Hispanic Black participants was highly (data not shown) consistent with previous studies [26,27]. The relatively high proportion of non-Hispanic Black participants in the stable obese group may explain the high incidence of hypertension in non-Hispanic Black participants.

This study had several strengths. We offered a unique opportunity with a large, nationally representative retrospective cohort to investigate the impact of weight loss from early-mid adulthood on incident hypertension risk. Thus, our results are broadly generalizable to the US population [13]. Additionally, our research was able to assess weight loss compared with weight maintenance while most previous studies focused on the effect of weight gain.

However, in the explanation of our present results, several potential limitations should also be considered. Firstly, we used self-reported weight for the analysis, instead of measured weight, which may lead to the misclassification of weight change status. However, high accuracy of self-reported weight as compared with measured weight has been validated in previous studies [28,29]. Secondly, BMI indicates both fat and lean mass; therefore, future studies using molecular markers that can distinguish between fat from lean mass would provide further insight. Thirdly, we could not obtain weight data at other time points between age 25 years to baseline, thus, we were unable to determine what time period the weight change occurred in this study. Future studies are warranted to explore the association between obesity trajectories and incident hypertension risk. Lastly, we could not adjust for family history of hypertension, alcohol consumption, physical activity or dietary factors because data on these variables at baseline were not collected. Therefore, our findings may partly reflect the changes in physical activity and diet patterns across the life course.

## 5. Conclusions

In summary, weight change from early to middle adulthood was an independent predictor for later-life hypertension. We found that weight loss was associated with lower hypertension risk compared with participants remained stable obese. Those who lost weight had a higher hypertension risk compared with those who maintained a normal weight throughout their adulthood. The findings from the national sample highlight the importance of monitoring weight changes and reducing BMI by lifestyle modifications, and they may be helpful in the primary prevention of hypertension. Finally, further prospective studies that investigate the association of weight loss with hypertension risk are warranted.

## Figures and Tables

**Table 1:** Characteristics of the National Health and Nutrition Examination Survey (NHANES) study participants according to weight change patterns from early to middle adulthood, 1999–2016 ^a^.

Characteristics	Stable Normal	Maximum Overweight	Non-Obese to Obese	Obese to Non-Obese	Stable Obese	Total
**Participants**	6063 (44.0)	5240 (35.1)	2345 (15.1)	149 (0.9)	745 (4.9)	14542
**Age, mean (SD ^b^), years ^c^**	41.5 (0.17)	43.1 (0.17)	44.6 (0.25)	39.9 (0.65)	39.3 (0.44)	42.4 (0.13)
**Female**	3678 (64.5)	2073 (38.5)	1152 (47.9)	60 (43.3)	371 (46.5)	7334 (51.8)
**Race/ethnicity**						
Mexican American	802 (4.6)	1046 (7.1)	516 (8.1)	40 (9.0)	128 (7.4)	2532 (6.2)
Non-Hispanic White	2869 (74.3)	2331 (74.1)	1039 (74.4)	69 (74.1)	321 (72.2)	6629 (74.2)
Non-Hispanic Black	1069 (8.1)	952 (8.5)	477 (9.4)	25 (9.0)	206 (14.1)	2729 (8.7)
Others	1323 (12.9)	911 (10.3)	313 (8.1)	15 (8.0)	90 (6.4)	2652 (10.9)
**Education ^d^**						
Less than high school	1360 (13.8)	1355 (15.1)	630 (15.6)	59 (22.8)	190 (14.8)	3594 (14.7)
High school or equivalent	1289 (20.8)	1151 (23.1)	543 (24.4)	27 (22.0)	180 (24.8)	3190 (22.3)
College or above	3409 (65.4)	2732 (61.8)	1171 (60.0)	63 (55.2)	374 (60.5)	7749 (63.0)
**Family income-poverty ratio level ^d^**						
0~1.3	1371 (14.6)	1205 (14.2)	591 (16.5)	54 (26.1)	213 (20.3)	3434 (15.1)
~1.85	615 (7.6)	547 (7.9)	266 (9.8)	20 (10.6)	91 (10.5)	1539 (8.2)
~3	894 (14.6)	795 (15.8)	405 (18.1)	18 (9.9)	117 (17.3)	2229 (15.6)
>3	2647 (63.3)	2251 (62.2)	886 (55.6)	52 (53.4)	263 (51.9)	6099 (61.1)
**Ever Smoker ^c^**	2790 (47.1)	2473 (48.3)	1100 (48.7)	105 (69.6)	308 (42.7)	6776 (47.7)
**Body mass index, mean (SD)**						
At age 25 years	20.7 (0.04)	23.8 (0.05)	25.1 (0.09)	33.9 (0.47)	34.7 (0.25)	23.3 (0.05)
At baseline	22.2 (0.03)	27.1 (0.03)	33.7 (0.11)	26.7 (0.29)	38.4 (0.35)	26.5 (0.06)
**Absolute weight change mean (SD), kg**	3.6 (0.09)	8.4 (0.15)	22.7 (0.41)	20.7 (1.73)	9.6 (1.00)	8.2 (0.12)

^a^ All estimates accounted for complex survey design; data are expressed as No. (%) unless otherwise indicated. ^b^ SD, Standard Deviation. ^c^ At start of follow-up. ^d^ At end of follow-up.

**Table 2:** Hazard ratios (HRs) and 95% confidence intervals (CIs) of incident hypertension with weight change patterns across adulthood ^a^.

Weight Change Patterns ^b^	Incident Hypertension n/(Person-Years)	Model 1 ^c^		Model 2 ^d^	
		HR (95% CI)	*p*	HR (95% CI)	*p*
**Stable Normal (reference)**	863/57,452	1.00		1.00	
Maximum Overweight	1197/47,644	1.76 (1.55, 2.00)	<0.001	1.76 (1.55, 2.00)	<0.001
Non-obese to Obese	838/19,826	2.98 (2.66, 3.33)	<0.001	2.93 (2.62, 3.28)	<0.001
Obese to Non-obese	29/1398	2.10 (1.26, 3.50)	0.005	1.97 (1.17, 3.31)	0.011
Stable Obese	250/6320	3.37 (2.79, 4.08)	<0.001	3.28 (2.71, 3.96)	<0.001
**Stable Obese (reference)**	250/6320	1.00		1.00	
Stable Normal	863/57,452	0.30 (0.25, 0.36)	<0.001	0.31 (0.25, 0.37)	<0.001
Maximum Overweight	1197/47,644	0.52 (0.43, 0.63)	<0.001	0.54 (0.44, 0.65)	<0.001
Non-obese to Obese	838/19,826	0.88 (0.72, 1.08)	0.219	0.89 (0.73, 1.09)	0.260
Obese to Non-obese	29/1398	0.62 (0.37, 1.05)	0.073	0.60 (0.35, 1.02)	0.059

^a^ All estimates accounted for complex survey designs. ^b^ Stable normal (BMI <25.0 at both timepoints), maximum overweight (BMI, 25.0–29.9 at either timepoint but not ≥30.0 at the other timepoint), non-obese to obese (BMI < 30.0 at younger age and ≥30.0 later), obese to non-obese (BMI ≥ 30.0 at younger age and <30.0 later), and stable obese (BMI ≥ 30.0 at both timepoints). ^c^ Model 1: adjusted for sex, baseline age, race/ethnicity. ^d^ Model 2: additionally adjusted for family income, education, smoking status.

**Table 3:** Hazard ratios (HRs) and 95% confidence intervals (CIs) of incident hypertension with absolute weight change patterns across adulthood ^a^.

Weight Change Patterns	Incident Hypertension n/(Person-Years)	Model 1 ^b^		Model 2 ^c^	
		HR (95% CI)	*P*	HR (95% CI)	*P*
**Weight loss ≥ 2.5 kg (ref)**	153/7914	1.00		1.00	
Weight change within 2.5 kg	574/36,666	0.71 (0.54, 0.92)	0.010	0.98 (0.76, 1.26)	0.868
2.5 kg ≤ Weight gain < 10 kg	920/45,218	1.02 (0.79, 1.31)	0.886	1.46 (1.14, 1.87)	0.003
10 kg ≤ Weight gain < 20 kg	851/27,153	1.49 (1.19, 1.87)	<0.001	2.09 (1.67, 2.60)	<0.001
Weight gain ≥ 20 kg	679/15,689	2.02 (1.56, 2.61)	<0.001	2.64 (2.07, 3.37)	<0.001
*P* for Trend ^d^		1.35 (1.30, 1.40)	<0.001	1.36 (1.31, 1.41)	<0.001

^a^ All estimates accounted for complex survey designs. ^b^ Model 1: adjusted for sex, baseline age, race/ethnicity. ^c^ Model 2: additionally adjusted for family income, education, smoking status, and BMI at 25 years. ^d^ For the test of trend, we calculated the association with incident hypertension by treating the categories of weight change patterns as ordinal variables.

## Supplementary Materials

The following are available online at https://www.mdpi.com/2072-6643/12/9/2622/s1, Figure S1: Survival analysis study design: weight change and onset hypertensions; Figure S2: A flow chart of inclusion and exclusion of study participants; Table S1: Hazard ratios (95% confidence intervals) of incident hypertension with BMI status at baseline in the NHANES 1999–2016; Table S2. Hazard ratios (95% CIs) of incident hypertension with weight change patterns stratified by BMI at 25 years; Table S3. Hazard ratios (95% CIs) of incident hypertension with weight change patterns across adulthood in NHANES 1999–2016.
