# Supplementary material for: Association of Weight Loss from Early to Middle Adulthood and Incident Hypertension Risk Later in Life
Source: Nutrients. 2020 Aug 28;12(9):2622. doi: 10.3390/nu12092622 (PMC7551896; doi:10.3390/nu12092622)
Supplement: Supplementary file 1 [file nutrients-12-02622-s001.pdf]

Supplemental Figure

Figure S1. Survival analysis study design: weight change and onset hypertensions.

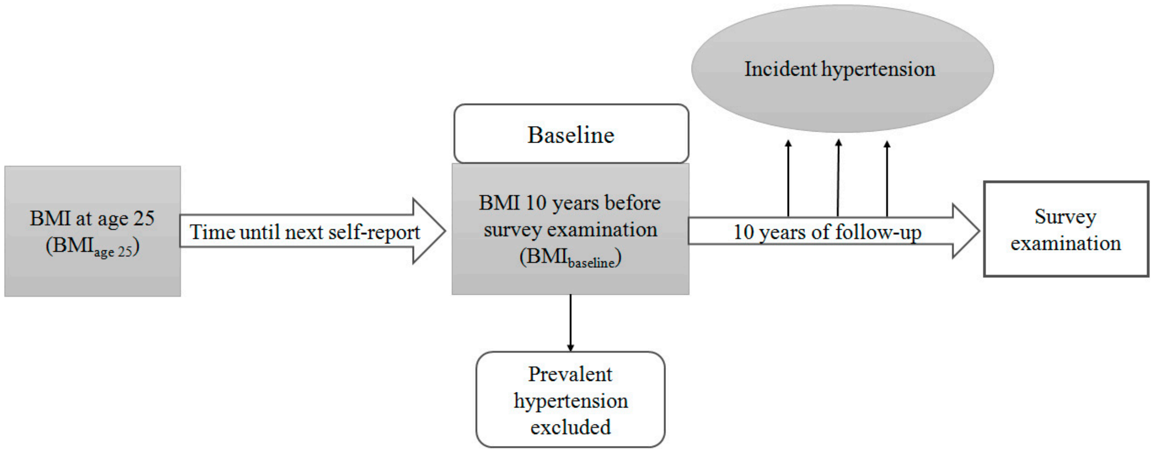

Figure S2. A flow chart of inclusion and exclusion of study participants.

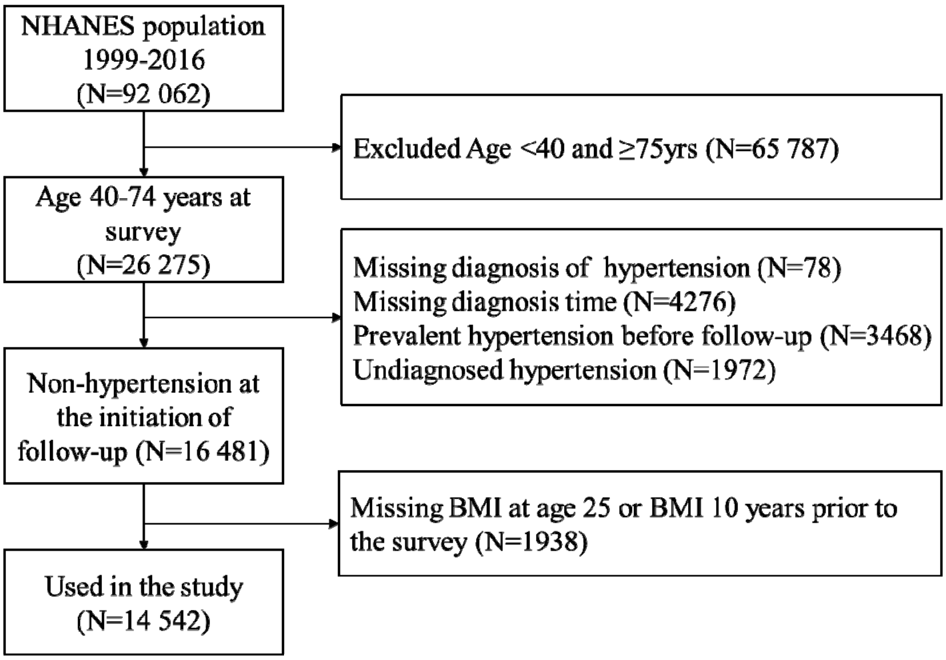

Table S1. Hazard ratios (95% confidence intervals) of incident hypertension with BMI status at baseline in the NHANES 1999-2016 [a]

Weight Patterns [b]	Model 1 [c]		Model 2 [d]	
	HR (95% CI)	*P*	HR (95% CI)	*P*
Underweight	0.95 (0.68, 1.33)	0.752	0.92 (0.61, 1.37)	0.673
**Normal (ref)**	1.00		1.00	
Overweight	1.76 (1.54, 2.02)	<0.001	1.80 (1.58, 2.05)	<0.001
Class 1 obesity	2.70 (2.39, 3.04)	<0.001	2.77 (2.42, 3.16)	<0.001
Class 2 obesity	3.59 (2.97, 4.35)	<0.001	3.31 (2.70, 4.07)	<0.001
Class 3 obesity	3.90 (3.26, 4.66)	<0.001	3.72 (3.05, 4.54)	<0.001
P for Trend [e]	1.46 (1.41, 1.51)	<0.001	1.44 (1.39, 1.50)	<0.001

[a] All estimates accounted for complex survey designs.

[b] We categorized BMI variables into six groups: underweight (<18.0) and normal weight (18.0-24.9), overweight (25.0-29.9), Class 1 obesity (30.0-34.9), Class 2 obesity (35.0-39.9), and Class 3 obesity (≥40).

[c] Model 1 : adjusted for baseline age, sex, race/ethnicity.

[d] Model 2 : additionally adjusted for education level, family income-poverty ratio level, smoking status.

[e] For test of trend, we calculated the association with incident hypertension by treating the categories of weight patterns as ordinal variables.

Table S2. Hazard ratios (95% CIs) of incident hypertension with weight change patterns stratified by BMI at 25 years [a]

Weight Change Patterns	Model 1 [b]		Model 2 [c]	
	HR (95% CI)	*P*	HR (95% CI)	*P*
**Underweight/Normal weight (BMI <25)**				
Weight loss ≥2.5 kg (ref)	1.00		1.00	
Weight change within 2.5 kg	0.83 (0.60, 1.15)	0.260	0.91 (0.66, 1.25)	0.558
Weight gain ≥2.5 kg and <10 kg	1.21 (0.87, 1.69)	0.253	1.36 (0.98, 1.89)	0.067
Weight gain ≥10 kg and <20 kg	1.79 (1.30, 2.45)	<0.001	2.00 (1.47, 2.73)	<0.001
Weight gain ≥20 kg	2.50 (1.81, 3.44)	<0.001	2.73 (1.98, 3.74)	<0.001
P for Trend [d]	1.41 (1.34, 1.49)	<0.001	1.41 (1.34, 1.49)	<0.001
**Overweight/Obese (BMI ≥25)**				
Weight loss ≥2.5 kg (ref)	1.00		1.00	
Weight change within 2.5 kg	0.94 (0.70, 1.28)	0.714	1.05 (0.78, 1.42)	0.751
Weight gain ≥2.5 kg and <10 kg	1.31 (0.92, 1.85)	0.130	1.51 (1.07, 2.14)	0.019
Weight gain ≥10 kg and <20 kg	1.75 (1.28, 2.39)	0.001	2.00 (1.48, 2.71)	<0.001
Weight gain ≥20 kg	2.05 (1.47, 2.86)	<0.001	2.24 (1.61, 3.10)	<0.001
P for Trend [d]	1.25 (1.17, 1.33)	<0.001	1.27 (1.19, 1.35)	<0.001

[a] All estimates accounted for complex survey designs.

[b] Model 1 : adjusted for baseline age, sex, race/ethnicity.

[c] Model 2 : additionally adjusted for education level, family income-poverty ratio level, smoking status.

[d] For test of trend, we calculated the association with incident hypertension by treating the categories of weight change patterns as ordinal variables.

Table S3. Hazard ratios (95% CIs) of incident hypertension with weight change patterns across adulthood in NHANES 1999-2016 [a]

Category	Weight Change Patterns	Incident Hypertension n/(person-years)	Model 1 [b]		Model 2 [c]	
			HR (95% CI)	*P*	HR (95% CI)	*P*
Category 1 [d]						
	**Stable Normal (ref)**	1560/64422	1.00		1.00	
	Maximum Overweight	1888/54554	1.43 (1.31, 1.57)	<0.001	1.43 (1.41, 1.57)	<0.001
	Non-obese to Obese	1123/22676	2.15 (1.95, 2.36)	<0.001	2.12 (1.93, 2.34)	<0.001
	Obese to Non-obese	48/1588	1.45 (0.94, 2.24)	0.091	1.38 (1.17, 3.31)	0.147
	Stable Obese	337/7190	2.42 (2.06, 2.83)	<0.001	2.37 (2.02, 2.77)	<0.001
	**Stable Obese (ref)**	337/7190	1.00		1.00	
	Stable Normal	1560/64422	0.41 (0.35, 0.48)	<0.001	0.42 (0.36, 0.49)	<0.001
	Maximum Overweight	1888/54554	0.59 (0.50, 0.70)	<0.001	0.61 (0.52, 0.71)	<0.001
	Non-obese to Obese	1123/22676	0.89 (0.75, 1.05)	0.175	0.90 (0.76, 1.06)	0.205
	Obese to Non-obese	48/1588	0.60 (0.39, 0.93)	0.024	0.59 (0.37, 0.92)	0.019
Category 2 [e]						
	**Weight loss ≥2.5 kg (ref)**	261/8994	1.00		1.00	
	Weight change within 2.5 kg	1011/41036	0.83 (0.68, 1.01)	0.068	1.04 (0.84, 1.28)	0.717
	Weight gain ≥2.5 kg and <10 kg	1532/51338	1.04 (0.86, 1.26)	0.676	1.36 (1.10, 1.68)	0.005
	Weight gain ≥10 kg and <20 kg	1247/31113	1.35 (1.13, 1.60)	0.001	1.77 (1.47, 2.15)	<0.001
	Weight gain ≥20 kg	905/17949	1.71 (1.41, 2.08)	<0.001	2.08 (1.68, 2.56)	<0.001

[a] All estimates accounted for complex survey designs. Hypertension cases including those who had elevated blood pressure at survey examination.
[b] Model 1 :adjusted for baseline age, sex, race/ethnicity.
[c] Model 2 : additionally adjusted for education level, family income-poverty ratio level, smoking status. In the Category 2 analyses, we also included BMI at the age 25 years as potential confounders in model 2.
[d] Stable Normal pattern (BMI <25.0 at both timepoints), Maximum Overweight pattern (BMI 25.0-29.9 at either timepoint but not ≥30.0 at the other timepoint), Non-obese to Obese pattern (BMI <30.0 at younger age and ≥30.0 later), Obese to Non-obese pattern (BMI ≥30.0 at younger age and <30.0 later), and Stable Obese (BMI ≥30.0 at both timepoints).
[e] Absolute weight change: weight loss of at least 2.5 kg, weight change within 2.5 kg, weight gain of at least 2.5 kg but less than 10.0 kg, weight gain of at least 10 kg but less than 20.0 kg, and weight gain of at least 20.0 kg.
